# Insights into the *Paulownia Shan tong* (Fortunei × Tomentosa) Essential Oil and In Silico Analysis of Potential Biological Targets of Its Compounds

**DOI:** 10.3390/foods13071007

**Published:** 2024-03-26

**Authors:** Călin Jianu, Marius Mioc, Alexandra Mioc, Codruța Șoica, Alexandra Teodora Lukinich-Gruia, Gabriel Bujancă, Matilda Rădulescu

**Affiliations:** 1 Faculty of Food Engineering, University of Life Sciences “King Michael I” from Timisoara, Calea Aradului 119, RO-300645 Timișoara, Romania; calin.jianu@gmail.com; 2 Faculty of Pharmacy, “Victor Babes” University of Medicine and Pharmacy, 2nd Eftimie Murgu Square, RO-300041 Timisoara, Romania; marius.mioc@umft.ro (M.M.); alexandra.petrus@umft.ro (A.M.); codrutasoica@umft.ro (C.Ș.); 3 OncoGen Centre, County Hospital “Pius Branzeu”, Blvd. Liviu Rebreanu 156, RO-300736 Timisoara, Romania; alexandra.gruia@hosptm.ro; 4 Faculty of Medicine, “Victor Babes” University of Medicine and Pharmacy, 2nd Eftimie Murgu Square, RO-300041 Timișoara, Romania; radulescu.matilda@umft.ro

**Keywords:** *Paulownia Shan tong* (Fortunei × Tomentosa), essential oils, chemical composition, antioxidant activity, molecular docking

## Abstract

The volatile composition of *Paulownia Shan tong* (Fortunei × Tomentosa) essential oil isolated by steam distillation (yielding 0.013% *v*/*w*) from flowers (forestry wastes) was investigated by gas chromatography–mass spectrometry. Thirty-one components were identified, with 3-acetoxy-7, 8-epoxylanostan-11-ol (38.16%), β-monoolein (14.4%), lycopene, 1,2-dihydro-1-hydroxy- (10.21%), and 9,12-octadecadienoic acid, 2-phenyl-1,3-dioxan-5-yl ester (9.21%) as main compounds. In addition, molecular docking was employed to identify potential protein targets for the 31 quantified essential oil components. Inhibition of these targets is typically associated with antibacterial or antioxidant properties. Molecular docking revealed that six of these components, namely, 13-heptadecyn-1-ol, ascabiol, geranylgeraniol, anethole, and quinol dimethyl ether, outperformed the native ligand (hypoxanthine) of xanthine oxidase in terms of theoretical binding affinity, therefore implying a significant in silico inhibitory potential against xanthine oxidase. These findings suggest that the essential oil extracted from *Paulownia Shan tong* flowers could be valuable for developing protein-targeted antioxidant compounds with applications in the food, pharmaceutical, and cosmetic industries.

## 1. Introduction

The utilization of natural volatile products holds considerable importance in various domains, including the environment, health, and pharmaceutical industries [1]. The application of these materials offers a practical resolution to environmental issues by decreasing dependence on artificial substitutes and mitigating ecological consequences through their biodegradability and organic origin [2]. Volatile compounds of natural origin are also of significant importance in the food industry as they contribute to the development of flavors, preservation, and sensory enhancement. These compounds play a crucial role in enhancing the variety and quality of food products while also satisfying the demands of customers for natural and healthy alternatives [3]. Within the pharmaceutical sector, these products assume a crucial function, encompassing both conventional remedies and modern therapies, thereby showcasing their adaptability and capacity to enhance human well-being [4]. A diverse array of plant species serves as a substantial reservoir of essential oils that encompass these natural volatile compounds. Currently, extensive research is being conducted to extract essential oils from various plant species. Various methods, such as chemical, computational, and biological approaches, are employed to analyze oils and their constituent compounds. These methods are utilized to explore the potential applications of these oils in the pharmaceutical industry [5,6,7]. The growing use of naturally occurring volatile products embodies a practical and comprehensive strategy for attaining sustainability goals while simultaneously addressing the varied requirements of society.

*Paulownia* is a member of the *Paulownia* genus (*Scrophulariaceae* family), native to China and Southeast Asia [8,9,10]. The genus *Paulownia* (*Paulowniaceae* family) consists of nine species such as *Paulownia fortunei* (*P. fortunei*), *Paulownia elongata* (*P. elongata*), *Paulownia catalpifolia* (*P. catalpifolia*), *Paulownia albiphloea* (*P. albiphloea*), *Paulownia tomentosa* (*P. tomentosa*), *Paulownia australis* (*P. australis*), *Paulownia kawakamii* (*P. kawakamii*), *Paulownia fargesii* (*P. fargesii*), and *Paulownia taiwaniana* (*P. taiwaniana*) [11,12,13]. In addition, several hybrids have also been created, such as the *Shan tong* hybrid, resulting from the cross between *P. tomentosa* and *P. fortune*, which has recently started to be cultivated in Romania as high-quality timber plantations [14]. *Paulownia* is widely recognized as a highly utilized medicinal plant due to its extensive utilization in traditional medicine, particularly within the context of Chinese traditional medicine. The investigation into the chemical components of the *Paulownia* genus commenced in the early 1930s. The field in question was initially explored by Japanese researchers. The isolation of glycoside compounds from the bark and leaves of *Paulownia* was achieved for the first time in 1931. Subsequently, additional categories of compounds were isolated from various species of *Paulownia* [11].

Usually, *Paulownia* is primarily produced for its wood and has substantial economic benefits in manufacturing furniture, airplanes, and musical instruments [15,16,17]. However, the abundant *Paulownia* flowers are viewed as a by-product and are usually dumped and left to rot into the soil, thus restricting their utilization [18,19]. In recent decades, research on the chemical composition, health benefits, and use of *Paulownia* flowers has made significant progress, leading to its recognition as a new source of bioactive principles. For example, the essential oil (EO) extracted from *P. tomentosa* flowers harvested in Egypt demonstrated antibacterial properties that suggested potential application in the food and pharmaceutical industries [20]. Zhang et al. (2019) showed that the nano-encapsulated *P. tomentosa* flowers EO blended with chitosan protect the surface of ready-to-cook pork chops by inhibiting lipid oxidation and microbiological growth, extending shelf-life and the quality properties during refrigerated storage [21]. Furthermore, Ferdosi et al. (2021) reported that EO isolated from *P. fortunei* flowers possessed antibacterial and antifungal properties [22].

On the other hand, there are some members of this genus that require additional research studies. According to the review of the relevant literature, there are no previous reports that outline the chemical composition of the essential oil that was obtained through steam distillation from *P. Shan tong* flowers. In light of these considerations, the objectives of our investigation were as follows: (a) to conduct a gas chromatography–mass spectrometry (GC–MS) analysis of the chemical composition of *P. Shan tong* essential oil (PStEO); and (b) to conduct a docking-based in silico identification of potential biological targets for the volatile compounds extracted from *P. Shan tong* flowers.

## 2. Materials and Methods

### 2.1. Plant Material Collection

*P. Shan tong* flowers were collected manually, at the maximum flowering stage, in April 2021 from S.C. PP Pepiniera și Plantatie S.R.L., Izvin village, Timiș County, Romania (GPS coordinates 45.8014° N, 21.4602° E), from a plantation that was founded in 2017 with plant material, which originated (lot no. 20170401) from the Shaanxi region of China. A voucher was deposited in the Herbarium of the Faculty of Agronomy, University of Life Sciences “King Michael I” from Timisoara.

### 2.2. PStEO Extraction

The PStEO was obtained from the fresh flowers using the steam distillation method using a Craveiro-type apparatus following the procedure previously described by Jianu et al. [23]. The steam and vaporized oil were condensed into liquid form by a condenser and collected in a water-cooled oil receiver to decrease the formation of artifacts due to overheating [24,25]. Afterward, the PStEO was recovered from the receiver with a sterile syringe, dried on anhydrous sodium sulfate (Sigma-Aldrich Chemie GmbH, Taufkirchen, Germany), and kept at −18 °C in sealed vials for future investigation (yielding 0.013% *v*/*w*).

### 2.3. PStEO GC–MS Analysis

The PStEO was analyzed on a gas chromatograph HP6890 and a mass spectrometer HP5973 (ionization energy: 70 eV) (Agilent Technologies, Santa Clara, CA, USA). The PStEO sample was diluted in hexane, and 1 μL was injected manually in splitless mode on a Br-5MS capillary column (5% Phenyl-arylene-95% Dimethylpolysiloxane, 30 m length, 0.25 mm internal diameter, 0.25 μm film thickness) (Bruker, Billerica, MA, USA). Helium was employed as carrier gas at a 1 mL/min flow rate. The oven temperature was held at 50 °C for 3 min and then raised by 6 °C/min to 300 °C. MS source and Quad temperatures were set at 230 °C and 150 °C, respectively. The compounds were scanned in a weight range between 50 to 550 amu. The calculation of retention indices (RI) was performed for all components that were injected under identical conditions as the samples by utilizing a reference set of C_8_-C_20_ n-alkanes (Sigma-Aldrich Chemie GmbH, Taufkirchen, Germany). The components of PStEO were identified by comparing their RIs with those of n-alkanes and comparing them with the NIST2.0 library (software developed by the USA National Institute of Science and Technology NIST, Gaithersburg, MD, USA) and existing literature data [26].

### 2.4. Molecular Docking

The present study employed a previously reported method [27] to conduct molecular docking simulations. In summary, the optimization of docking targets was performed by utilizing the 3D crystallographic structure of proteins obtained from the RCSB Protein Data Bank [28], detailed in Table 1. The SDF (structure data format) structure files of the 31 components of the PStEO were obtained from the PubChem database [29]. Subsequently, these files were converted into PDBQT files (an enhanced version of the pdb format, specifically tailored to accommodate the necessary information for the protein-ligand docking software AutoDock 4) using Autodocktools [30]. Molecular docking was performed using the PyRx v0.8 virtual screening software developed by The Scripps Research Institute (La Jolla, CA, USA). As previously described, the docking calculations utilized Vina’s embedded scoring function [31]. Each target protein was subjected to docking, with the default number of conformers (8 per ligand structure). The structure of each native ligand (NL) for the proteins, listed in Table 1, was obtained from their corresponding PDB file and subsequently converted to the PDBQT format, following the earlier procedure. To validate our protocol, it was necessary to ensure that the root mean square deviation (RMSD) between the predicted docked and experimental pose of the native ligand did not surpass a threshold of 2 Å for each case. The grid box parameters selection was made to precisely align with the active binding domain, as indicated in Table 1. The software computed the ΔG binding energy values (expressed in kcal/mol) as docking scores for every docked molecule. The protein–ligand binding interactions were investigated using Accelrys Discovery Studio 4.1, a software developed by Dassault Systems BIOVIA (San Diego, CA, USA).

## 3. Results and Discussions

### 3.1. Chemical Composition of the PStEO

**Figure 1:**
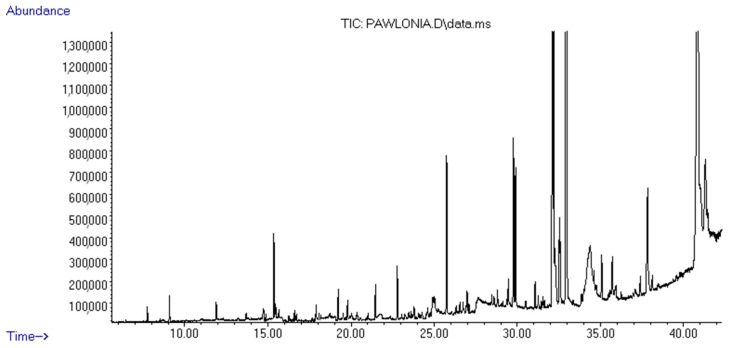
Gas chromatogram of the essential oil of the *Paulownia Shan tong* (Fortunei × Tomentosa) flowers.

The isolation yield of the PStEO obtained from *P. Shan tong* flowers was 0.013% *v*/*w*. The GC–MS of PStEO identified thirty-one compounds, summarized in Table 2, according to their elution order on a Br-5MS capillary column. The gas chromatogram obtained for the extracted PstEO is depicted in Figure 1. The main compounds were 3-acetoxy-7, 8-epoxylanostan-11-ol (38.16%), β-monoolein (14.4%), lycopene, 1,2-dihydro-1-hydroxy (10.21%) and 9,12-octadecadienoic acid, 2-phenyl-1,3-dioxan-5-yl ester (9.21%) (Table 1). Other compounds identified were ethyl iso-allocholate (4.71%), glycidol stearate (4.51%), geranyl linalool (3.93%), heptacosane (3.71%), and geranyl acetone (1.83%), respectively. Wang et al. (2005) conducted a study on the oil extracted from *P. tomentosa* flowers using steam distillation. The researchers found that the main compounds present in the extracted oil were 2-methoxy-3-(2-propenyl)-phenol (6.14%), 1,2,4-trimethoxybenzene (8.34%), and benzyl alcohol (13.28%) [32]. During the present study, it was observed that 1,2,4-trimethoxybenzene remained present in the essential oil, albeit not constituting a significant component (0.21%). Conversely, the other two aforementioned major compounds were not detected. Zhang et al. (2010) identified the major constituents in the oil derived from *P. elongata* flowers [33]. The lead major compounds were (E)-3, 7-dimethyl-1, 3, 6-octatriene, 1-octen-3-ol, methyl benzoate, 4-dimethoxy-benzene, methyl salicylate, and 1-methoxy-4-(1-propenyl)-benzene [33]. The only compound in common with this study was 1,4-dimethoxybenzene (quinol dimethyl ether), which was detected in our case as a minor component accounting for 0.53% of the total extracted essential oil. In some cases, major components of other *Paulownia*-extracted essential oils were not detectable in our quantified essential oil. One such study, reported by Ferdosi et al. (2021) [22], investigated the flower oil obtained from *P. fortune.* Nerolidol was found to account for 82.81% of the composition of *P. fortunei* flower oil, according to a study conducted by Ferdosi et al. (2021) [22]. Octadecane was found to account for 4.77% of the composition, and pentacosane was found to account for 3.95%. As observed, none of these compounds were found in the extracted PStEO [22]. These results suggest that PStEO isolated from *Paulownia* flower species possess a high chemical composition diversity and highlight the opportunity to systematically investigate genus species to identify novel sources of biologically active compounds with future applications in the food and pharmaceutical industries.

### 3.2. Molecular Docking

Our study aimed to determine whether the PStEO had the potential to exhibit antioxidant or antibacterial properties in silico based on a molecular docking approach. This theory was based on the possibility that the PStEO components can inhibit both target proteins involved in bacterial metabolism, typically targeted by antibiotics or chemotherapeutics, and proteins involved in oxidative stress triggered by the production of reactive oxygen species. For the antibacterial activity in silico assessment, the list of target proteins evaluated consists of DNA gyrase, D-alanine: D-alanine ligase (DDl1), dihydrofolate reductase (DHFR), DNA gyrase subunit, B and penicillin-binding protein 1a (PBP1a). Molecular docking results of the 31 PStEO components against the proteins mentioned above are presented in Table 3.

**Figure 2:**
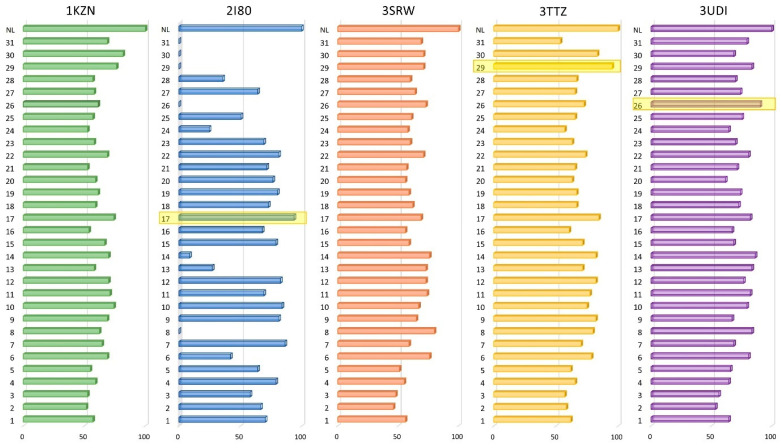
Graphical representation of the obtained docking score values related to protein targets involved in antimicrobial activity, corresponding to the 31 PStEO constituents; docking score values are calculated as a percentage of the native ligand’s docking score of each target protein and are plotted as series in a bar chart; compounds with scores above 90% are highlighted in yellow.

The in silico technique was employed to find protein targets that could be inhibited by various PStEO constituents or, at the very least, by the main components. According to docking results, in the case of all five proteins under investigation, no docked compound outperformed the native ligand in any situation. Since each protein has unique binding site characteristics and native ligands generate different docking scores, resulting in different control values for each score set, it can be challenging to determine correlations between docking scores of the same ligand against various proteins. To make up for these shortcomings, we expressed each docking score as a percentage of the score achieved by the corresponding native ligand (which was set at 100%). A bar chart was used to represent these percentages. Compounds with a calculated negative percentage were set at 0%. The final score should show a more obvious tendency for the 31 PStEO components to equal or outperform the NL’s score (100%). Figure 2 shows the modified docking scores used to predict the antimicrobial activity of the PStEO components. No discernible pattern exists where most compounds reach the NL docking score. Only three compounds in three different cases scored in the 90–100% range. These were compound 17 (ascabiol) docked in DDl1 (2I80), compound 26 (cis-9,12-octadecadienoic acid, 2-phenyl-1,3-dioxan-5-yl ester) docked in PBP1a (3UDI), and compound 29 (ethyl iso-allocholate) docked in DNA gyrase subunit B (3TTZ), where the latter scored the closest to the native ligand (95%). However, given that most compounds in each case scored below 90%, we can infer that the PStEO would not be an appropriate source for antibacterial potent compounds.

**Figure 3:**
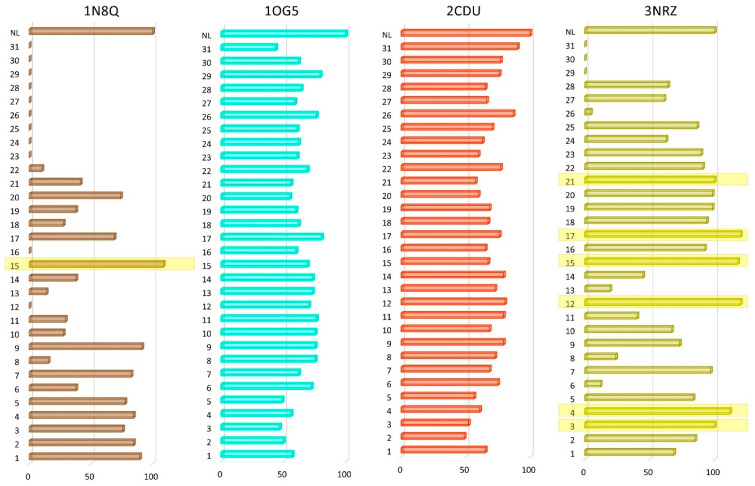
Graphical representation of docking score values related to protein targets involved in antimicrobial activity, corresponding to the PStEO constituents; docking score values are calculated as a percentage of the native ligand’s docking score of each target protein and are plotted as series in a bar chart; compounds with scores equal and above 100% are highlighted in yellow.

The in silico protein-targeted antioxidant effect of the 31 PStEO constituents was determined using the methodology described above. Four proteins were selected as our targets based on their involvement in the production of ROS. These targets include CYP2C9, NADPH-oxidase, Xanthine oxidase, and Lipoxygenase. Table 4 shows the docking results obtained following the in silico evaluation of the 31 PStEO compounds against the abovementioned four targets. According to the recalculation of the docking scores as docking percentages of the NL score (Figure 3), despite some 0% cases, six compounds (3, 4, 12, 15, 17, and 21) scored the same or above the NL, with the highest scorers being compound 17 (ascabiol, 122.4%), compound 12 (geranylgeraniol, 120.9%), and compound 15 (azulene, 117.9%). Compounds 19 (9-hexadecenoic acid) and 20 (2-hexadecanol) are two additional structures that are worth mentioning because they both scored nearly as high as the NL score (98.5% each). Interestingly, compound 15 outperformed the NL when docked in the lipoxygenase active site (1N8Q).

**Figure 4:**
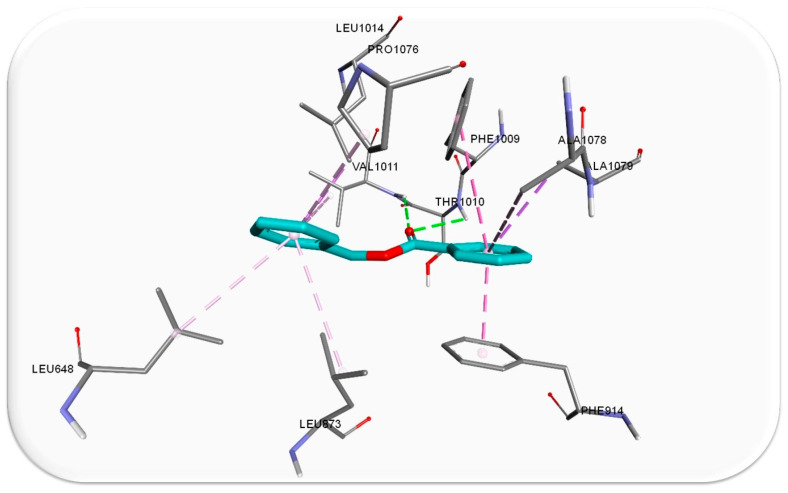
Docked compound ascabiol interacts with neighboring amino acids (grey sticks) through hydrophobic interactions (pink/purple dotted lines) and hydrogen bonds (green dotted lines) in the active site of xanthine oxidase (3NRZ).

To our knowledge, no existing literature currently investigates the inhibitory impact of ascabiol (benzylbenzoate) on xanthine oxidase. A recent study has demonstrated that three benzoate esters, which share a similar benzylbenzoate structure, exhibited significant inhibitory potential against xanthine oxidase in both in vitro and in silico experiments. Notably, these compounds outperformed the well-established inhibitor allopurinol [35]. Regarding the formed interactions between the ligand, ascabiol, and xanthine oxidase, it can be observed that the compound primarily forms hydrophobic interactions, as depicted in Figure 4. The compound also interacts through a pair of hydrogen bonds, one with Val 1011 (valine) and another with Thr 1010 (threonine). The latter interaction occurs when xanthine oxidase binds to its natural substrate, hypoxanthine [36].

## 4. Conclusions

This study reports the chemical composition of PStEO and in silico identification of potential biological targets of its volatile compounds for the first time. The GC–MS investigations indicated the presence of a unique molecule 3-acetoxy-7, 8-epoxylanostan-11-ol (38.16%), the main compound of PStEO. In addition, the results obtained from molecular docking analysis indicated that six PStEO components exhibited significant in silico inhibitory potential against xanthine oxidase. These findings suggest that the PStEO could be valuable for developing protein-targeted antioxidant compounds with applications in the food, pharmaceutical, and cosmetic industries. Still, further investigations are required to explore the biological properties of PStEO and elucidate the chemical composition of its oil.

## Figures and Tables

**Table 1:** Docking parameters used in the docking-based in silico assessment of the 31 PStEO components.

Protein	PDB ID	Native Ligand Name/Chemical Structure	Grid Box Center Coordinates and Size (Å)
*Escherichia coli* DNA gyrase	1KZN	Clorobiocin 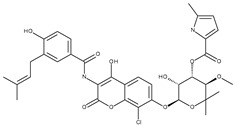	Grid center coordinates: x = 18.0206 y = 30.6795 z = 35.3259 Grid size along the axes: x = 14.0835 Å y = 15.9180 Å z = 19.3350 Å
*Staphylococcus aureus* D-alanine: D-alanine ligase (DDl1)	2I80	3-chloro-2,2-dimethyl-N-[4-(trifluoromethyl)phenyl]propenamide 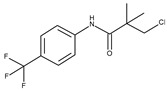	Grid center coordinates: x = 35.8534 y = 3.9116 z = 25.8999 Grid size along the axes: x = 10.2217 Å y = 12.8046 Å z = 10.2436 Å
*Staphylococcus aureus* Dihydrofolate reductase (DHFR)	3SRW	7-(2-ethoxynaphthalen-1-yl)-6-methylquinazoline-2,4-diamine 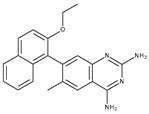	Grid center coordinates: x = −4.5959 y = −29.9973 z = 6.2901 Grid size along the axes: x = 10.7001 Å y = 15.8621 Å z = 14.9382 Å
*Staphylococcus aureus* DNA gyrase subunit B	3TTZ	2-[(3S,4R)-4-{[(3,4-dichloro-5-methyl-1H-pyrrol-2-yl)carbonyl]amino}-3-fluoropiperidin-1-yl]-1,3-thiazole-5-carboxylic acid 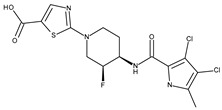	Grid center coordinates: x = 16.5842 y = −18.6283 z = 6.6255 Grid size along the axes: x = 17.6122 Å y = 10.7091 Å z = 8.5457 Å
*Acinetobacter baumannii* Penicillin-binding protein 1a (PBP1a)	3UDI	Penicillin G-open form 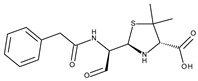	Grid center coordinates: x = 34.3158 y = −1.1961 z = 12.2776 Grid size along the axes: x = 11.8683 Å y = 10.7091 Å z = 12.2516 Å
Lipoxygenase	1N8Q	Protocatechuic acid 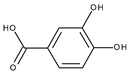	Grid center coordinates: x = 20.8705 y = 0.9784 z = 19.1589 Grid size along the axes: x = 9.4722 Å y = 6.7976 Å z = 10.6056 Å
CYP2C9	1OG5	S-Warfarin 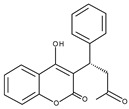	Grid center coordinates: x = −19.7808 y = 86.6496 z = 38.0890 Grid size along the axes: x = 11.6826 Å y = 11.6610 Å z = 10.9857 Å
NADPH-oxidase	2CDU	Adenosine-5’-diphosphate 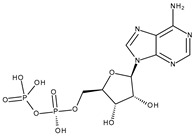	Grid center coordinates: x = 18.5709 y = −5.1763 z = −0.3019 Grid size along the axes: x = 17.8103 Å y = 15.7471 Å z = 15.5473 Å
Xanthine oxidase	3NRZ	Hypoxanthine 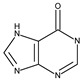	Grid center coordinates: x = 38.1287 y = 19.4742 z = 18.4338 Grid size along the axes: x = 10.6615 Å y = 10.8740 Å z = 13.6877 Å

**Table 2:** Organic volatile compounds of PStEO.

Common Name	RI_calc_^a^	RI_lit_^b^	Area %
Terpenes			
3-Carene	1029	1009	0.06
Cedrane	1444	1435	0.16
α-Bergamotene	1512	-	0.15
β-Bisabolene	1530	1547	0.25
β-Copaene	1535	-	0.15
Guaiazulene	1687	1772	0.47
Sub-totals (Class compounds %)			1.24
Terpenoids			
Globulol	1473	1584	0.39
Geranylgeraniol	1593	-	0.35
Caryophyllene oxide	1602	1578	0.12
Cadinol	1659	1679	0.69
Geranyl linalool	1981	2034	3.93
Sub-totals (Class compounds %)			5.48
Alcohols			
2,9-Octadecenyloxyethanol	1715	-	0.36
2-Hexadecanol	1885	1702	0.42
13-Heptadecyn-1-ol	1898	-	0.16
3-Acetoxy-7, 8-epoxylanostan-11-ol	2726	-	38.16
Sub-totals (Class compounds %)			39.1
Esters			
Ascabiol	1770	1760	0.82
Glycidol stearate	2179	-	4.51
Adipic acid, dioctyl ester	2244	-	0.71
9,12-Octadecadienoic acid, 2-phenyl-1,3-dioxan-5-yl ester	2295	-	9.21
Ethyl iso-allocholate	2577	-	4.71
Sub-totals (Class compounds %)			19.96
Others			
Nonanal	1092	1089	0.33
Quinol dimethyl ether	1158	1163	0.53
Anethole	1297	1301	0.41
1,2,4-trimethoxybenzene	1386	1377	0.21
Geranyl acetone	1468	1454	1.83
Phytone	1835	1838	1.25
9-Hexadecenoic acid	1869	1942	0.16
Heptacosane	2186	429	3.71
β-Monoolein	2299	-	14.4
3-Ethyl-5-(2-ethylbutyl)-octadecane	2441	-	1.16
Lycopene, 1,2-dihydro-1-hydroxy-	2749	4025	10.21
Sub-totals (Class compounds %)			34.2
Total (Class compounds %)			99.98

^a^ the retention index (RI_calc_) was calculated using a homologous series of n-alkanes C_8_–C_20_; ^b^ RI_lit_–RIs extracted from literature data [26,34].

**Table 3:** Docking scores of the 31 PStEO components against proteins targeted for the predicted antimicrobial activity.

Target PDB ID	1KZN	2I80	3SRW	3TTZ	3UDI
Docked EO component ID	Docking score. ∆G (kcal/mol)				
Native ligand	−9.3	−8.1	−10	−8.4	−7.3
1	−5.3	−5.7	−5.6	−5.2	−4.7
2	−4.8	−5.4	−4.6	−4.9	−3.9
3	−4.9	−4.7	−4.8	−4.8	−4.1
4	−5.5	−6.4	−5.5	−5.5	−4.7
5	−5.1	−5.2	−5.1	−5.2	−4.8
6	−6.4	−3.4	−7.6	−6.6	−5.9
7	−6	−7	−5.9	−5.9	−5
8	−5.8	1.20	−8	−6.7	−6.1
9	−6.4	−6.6	−6.5	−6.9	−4.9
10	−6.9	−6.8	−6.7	−6.3	−5.8
11	−6.6	−5.6	−7.4	−6.5	−6
12	−6.5	−6.7	−7.3	−6.9	−5.6
13	−5.4	−2.2	−7.3	−6	−6.1
14	−6.5	−0.7	−7.6	−6.9	−6.3
15	−6.2	−6.4	−5.9	−6	−5
16	−5	−5.5	−5.6	−5.1	−4.9
17	−6.9	−7.6	−6.9	−7.1	−6
18	−5.5	−5.9	−6.2	−5.6	−5.3
19	−5.7	−6.5	−5.9	−5.6	−5.4
20	−5.5	−6.2	−5.6	−5.3	−4.5
21	−4.9	−5.8	−5.7	−5.5	−5.2
22	−6.4	−6.6	−7.1	−6.2	−5.9
23	−5.4	−5.6	−6	−5.3	−5.1
24	−4.9	−2	−5.8	−4.8	−4.7
25	−5.3	−4.1	−6.1	−5.5	−5.5
26	−5.7	0.9	−7.3	−6.1	−6.6
27	−5.4	−5.2	−6.4	−5.5	−5.4
28	−5.3	−2.9	−6	−5.6	−5.1
29	−7.1	3.40	−7.1	−8	−6.1
30	−7.6	16.40	−7.1	−7	−5
31	−6.4	26.10	−6.9	−4.5	−5.8

**Table 4:** Docking scores of the 31 PStEO components against proteins targeted for the predicted antioxidant activity.

Target PDB ID	1N8Q	1OG5	2CDU	3NRZ
Docked EO component ID	Docking score, ∆G (kcal/mol)			
Native ligand	−5.8	−9.8	−9.2	−6.7
1	−5.2	−5.6	−6	−4.6
2	−4.9	−4.9	−4.5	−5.7
3	−4.4	−4.6	−4.8	−6.7
4	−4.9	−5.5	−5.6	−7.5
5	−4.5	−4.8	−5.2	−5.6
6	−2.2	−7.1	−6.9	−0.8
7	−4.8	−6.1	−6.3	−6.5
8	−0.9	−7.4	−6.7	−1.6
9	−5.3	−7.4	−7.3	−4.9
10	−1.6	−7.4	−6.3	−4.5
11	−1.7	−7.5	−7.3	−2.7
12	0.3	−6.9	−7.4	−8.1
13	−0.8	−7.2	−6.7	−1.3
14	−2.2	−7.2	−7.3	−3
15	−6.3	−6.8	−6.2	−7.9
16	1	−5.9	−6	−6.2
17	−4	−7.9	−7	−8.2
18	−1.6	−6.1	−6.2	−6.3
19	−2.2	−5.9	−6.3	−6.6
20	−4.3	−5.4	−5.5	−6.6
21	−2.4	−5.5	−5.3	−6.7
22	−0.6	−6.8	−7.1	−6.1
23	4	−6	−5.5	−6
24	10.40	−6.1	−5.8	−4.2
25	4.80	−6	−6.5	−5.8
26	17.40	−7.5	−8	−0.3
27	4.40	−5.8	−6.1	−4.1
28	5.90	−6.3	−6	−4.3
29	35.3	−7.8	−7	8.70
30	71.5	−6.1	−7.1	19.60
31	76.9	−4.3	−8.3	28.40

## Data Availability

The original contributions presented in this study are included in the article; further inquiries can be directed to the corresponding author.
